# Advancing Oxygen Evolution Catalysis with Dual-Phase Nickel Sulfide Nanostructures

**DOI:** 10.1021/acs.energyfuels.4c05182

**Published:** 2025-01-02

**Authors:** Neelakandan M Santhosh, Suraj Gupta, Vasyl Shvalya, Martin Košiček, Janez Zavašnik, Uroš Cvelbar

**Affiliations:** † Department of Gaseous Electronics (F6), Jožef Stefan Institute, Jamova cesta 39, 1000 Ljubljana, Slovenia; ‡ Jožef Stefan International Postgraduate School, Jamova cesta 39, SI-1000 Ljubljana, Slovenia; § Advanced Materials Department, Jožef Stefan Institute, Jamova 39, 1000 Ljubljana, Slovenia; ∥ Max-Planck-Institut für Nachhaltige Materialien, Max-Planck-Straße 1, 40237 Düsseldorf, Germany

## Abstract

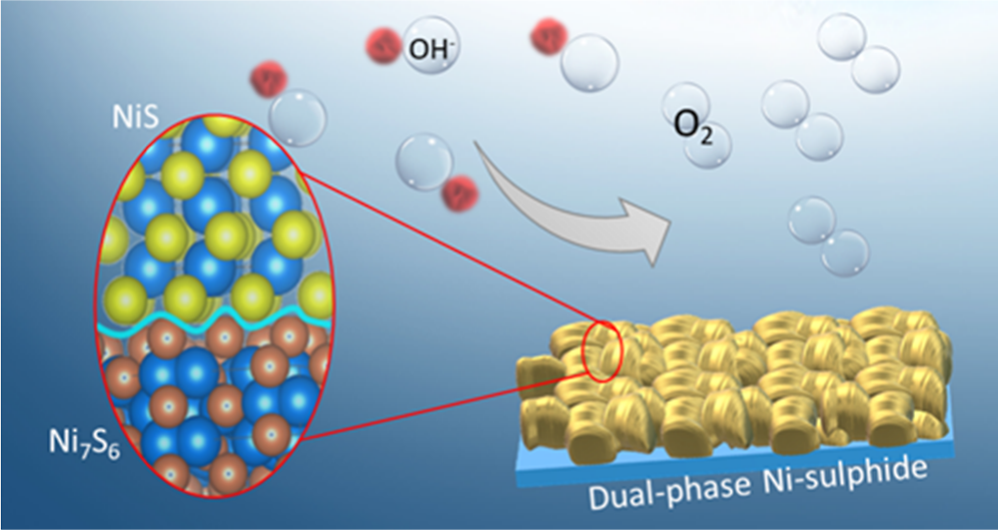

The production, conversion and storage of energy based on electrocatalysis, mainly assisted by oxygen evolution reaction (OER), plays a crucial role in alkaline water electrolyzers (AWEs) and fuel cells. Nevertheless, the insufficient availability of highly efficient catalyst materials at a reasonable cost that overcome the sluggish electrochemical kinetics of the OER is one of the significant obstacles. Herein, we report a fast and facile synthesis of vapor phase deposition of dual-phase nickel sulfide (Ni-sulfide) using low-temperature annealing in the presence of H_2_S and demonstrated as an efficient catalyst for OER to address the issues with sluggish electrochemical kinetics. The dual-phase Ni-sulfide structures consist of densely packed 10–50 μm microcrystals with 40–50 individual dual-phase layers, such as NiS and Ni_7_S_6_. As an electrocatalyst, the dual-phase Ni-sulfide exhibits excellent OER activity by achieving a current density of 10 mA/cm^2^ at an overpotential (η_10_) of 0.29 V and excellent electrochemical stability over 50 h. Besides, the Ni-sulfide displays considerable electrochemical robustness in alkaline conditions and forms OER-active Ni-oxide/hydroxide species during the process. Using an energy-efficient synthesis method, the fabricated unique crystalline nanodesign of dual-phase Ni-sulfide could open new pathways for the controlled synthesis of a high-efficiency group of electrocatalysts for a long-time stable electrochemical catalytic activity.

## 1 Introduction

Owing to the rising energy demand and the depletion of conventional fossil fuel resources, developing alternative sources to produce green and clean energy is an increasing area of research interest. Besides electrochemical energy storage devices, hydrogen (H_2_) is also considered a front-runner candidate for green energy conversion and storage due to its high calorific value and environmental friendliness.^1,2^ Electrochemical water splitting is one of the most efficient methods for producing green hydrogen. Nevertheless, the process is still challenging due to the kinetically slow oxygen evolution reaction (OER), necessitating more efforts to improve the OER rates.^3,4^ In acidic proton-exchange membrane water electrolyzers, Ir-based materials are used as OER electrocatalysts, but also challenging for upscaling due to their high cost and limited availability.^5,6^ Alkaline water electrolyzers (AWEs) overcome this challenge as they allow the use of non-noble metal electrodes.^7^ Ni-based catalysts are prominently used as anodes and cathodes in AWEs, but their low catalytic rates and stability are major issues that affect the overall electrolyzer efficiency.^4^

In this aspect, several studies explore the possibilities of designing advanced nanostructures based on transition metal derivatives as efficient electrocatalysts capable of replacing the current state-of-the-art materials in AWEs, especially for the OER.^3,8−11^ Designing and tailoring nanostructures with multifunctional features enables more active sites for catalytic activities and improves the charge transfer during the electrochemical reactions.^12^ Some of the most popular non-noble electrocatalysts belong to the class of metal oxides,^13−15^ phosphides,^16−18^ sulfides,^19,20^ borides^21,22^ and nitrides.^23,24^ From these, metal sulfides have been used in the last decades and are well-known for their bifunctional characteristics. For instance, MoS_2_ is one of the most widely researched catalysts of hydrogen evolution reaction (HER), with predicted properties close to that of Pt.^25,26^ Likewise, Ni and Co-sulfides are well-known HER and OER catalysts, showing excellent electrochemical performance.^13,27,28^ As Ni-based catalysts exhibit better stability in alkaline electrolytes, nickel sulfides are highly desired anode materials for AWEs. Nickel sulfides are expressed in various crystalline phases, such as NiS, NiS_2_, Ni_3_S_2_, and Ni_7_S_6_ and the electrochemical activity of the polymorphs are well explored.^29−31^ Besides the low cost, good electrical conductivity and easy synthesis approaches of nickel sulfides make them a front-runner among the different metal sulfide-based electrocatalysts. A promising strategy to go beyond the reported activities is incorporating multiple polymorphic phases in a single catalyst, which allows leveraging of the advantages of different phases. The heterojunction formed between the two phases can serve as the active adsorption sites for reactive intermediates, assisting in initiating and facilitating OER.^32,33^ Moreover, the polymorphs benefit from the compositional advantages and also feature different structural motifs to allow the formation of heterointerfaces.^34^ In several studies, different polymorphs of nickel sulfides have been combined to design highly efficient electrocatalysts.^30,34,35^ Also, it is shown that combining higher-order nickel sulfides with NiS could facilitate water dissociation and accelerate the electrochemical reaction.^36^ Thus, combining heterostructures with NiS as one polymorph could be a promising electrocatalyst for OER. Apart from the polymorphic nickel sulfide heterostructures, nickel sulfide-based catalysts have also been combined with various nanoarchitectures to improve the water-splitting performance. Several examples of similar strategies are reported in the form of MoS_2_/NiS_*x*_,^37,38^ CoS_*x*_/Ni_3_S_2_,^39^ CuS/NiS_*x*_^40^ and FeS/NiS_*x*_.^41^

Electrocatalytic activities are highly influenced by nanostructure morphology and structural organization, and the OER activity can be further improved through nanostructure design. In this regard, several methods were employed to design nickel sulfide-based nanostructures and their polymorphs with different geometries and orientations.^42,43^ Template-free hydrothermal synthesis, atomic layer deposition, chemical vapor deposition, and plasma deposition are various methods to design and develop nickel sulfide-based nanostructures.^30,44^ Even though hydrothermal synthesis has been known as a favorable approach for the fast design of nickel sulfide derivatives, the complexity of controlling the compositions is still a great challenge. Therefore, developing a fast approach to designing nickel sulfide-based nanostructures with single or multiphase structures would provide a new paradigm in the research for efficient electrocatalysts for green energy production.

Here, a fast and facile approach was demonstrated for directly fabricating hybrid dual-phase nickel sulfide nanostructures. The hybrid structure comprises the polymorphs of nickel sulfide, i.e., NiS and Ni_7_S_6_, which were directly grown on the nickel foil using a low-temperature sulfurization method. The Ni foil was directly treated in the presence of H_2_S gas at 200 °C for the direct growth of the hybrid electrocatalyst using vapor phase deposition. The dual-phase nanoarchitectures comprised of microcrystallites with sizes of 10–50 μm. As an electrocatalytic agent, the designed electrode exhibits excellent OER activity facilitated by the heterojunction between the two phases. At anodic potentials, the catalyst surface was observed to undergo a chemical transformation, forming OER active Ni-oxide/hydroxide species, which was investigated through several methods. The dual-phase catalyst grown on Ni foil showed considerable electrochemical robustness, proving its operational capability in alkaline conditions with an overpotential (η_10_) of 0.29 V and operational stability over 50 h. The demonstrated energy-efficient synthesis method and dual-phase nano design with unique crystalline structures could be a promising approach for the controlled synthesis of highly efficient metal sulfide-based electrocatalysts for sustainable energy production applications.

## 2 Experimental Section/Methods

### 2.1 Fabrication of Nickel Sulfide Electrodes

The nickel sulfide nanostructures were directly grown on the nickel substrate using a single-step low-temperature annealing technique. A nickel foil (0.25 mm thick, 99.5% metal basis, Fischer Scientific) cut into a 19 mm diameter disc (surface area 2.835 cm^2^) was used as the substrate. The disc substrate was cleaned and washed with acetone and placed in the heating zone of an 80 cm long tube furnace (OTF-1200X-II, MTI Corp.). The tube had a diameter of 8 cm and a heating zone of 45 cm. In the first step, the tube was vacuum sealed and pumped below 1 Pa. Later, H_2_S gas (99.5% purity) was fed into the system to elevate the pressure to 5 × 10^3^ Pa. Then, the sulfurization process was conducted at 200 °C for 2 h. Finally, the furnace system was cooled to room temperature, and the H_2_S gas was pumped out.

### 2.2 Characterization Techniques

#### 2.2.1 Morphology and Crystal Structure

The surface morphology of the nickel sulfide nanostructures was studied by a scanning electron microscope (SEM; Prisma E, Thermo Fisher Scientific Inc.) operating at 10 kV, equipped with an energy-dispersive X-ray spectrometer (EDS). The morphology and phase composition of the nanostructures were analyzed by a transmission electron microscope (TEM: JEM-2100, JEOL) operating at 200 keV equipped with a slow-scan CCD camera and an energy-dispersive X-ray spectrometer (EX-24063JGT, Jeol Inc.) and scanning transmission electron microscope (STEM, Talos, Thermo Fisher Scientific). For TEM analysis, the sulfide layer was detached from the Ni foil by bending the sample, and the flakes were further ground in a mortar, diluted in ethanol and sonicated for 30 min in an ultrasonic cleaner. The solution was transferred onto commercial Cu-supported holey carbon grids, which were mounted in low-background Be holder. The phase composition and crystal structure of the nickel sulfide structures were investigated by an X-ray diffractometer (D4 Endeavor, Bruker AXS GmbH), using K-alpha radiation operating at 30 mA, 40 kV. The spectra were recorded in the range of 2θ: 5–100° with a step size of 0.04° 2θ.

#### 2.2.2 Surface and Chemical Composition

Structural features of the synthesized nickel sulfide coating were analyzed by Raman spectroscopy (NTEGRA confocal Raman spectrometer, NT-MDT). The spectra were measured at four points on the sample surface to avoid uncertainties. An excitation wavelength of 488 nm was used for the spectra recording. Surface elemental analysis and chemical composition structures were characterized using X-ray photoelectron spectroscopy (XPS; PHI-TFA XPS spectrometer equipped with Al-monochromatic X-ray source at an energy of 1486.6 eV, Physical Electronics Inc.). Multipak Software was used to interpret the spectra and deconvolution of the peaks.

### 2.3 Electrochemical Measurements

The electrochemical measurements were performed in a 3-electrode assembly where bare and dual-phase Ni-sulfide-coated Ni foil substrates were used as the working electrode while graphite rod (3 mm diameter) and Hg/HgO (saturated KOH) acted as the counter and reference electrodes, respectively. The average mass loading of the nickel sulfide films was estimated as 0.8–1 mg/cm^2^. 1 M KOH (>85% pure) was used as the electrolyte and was purged with nitrogen gas for 10–15 min before use. All the reported potentials are converted for the reversible hydrogen electrode (RHE) unless stated otherwise. The conversion was done using the Nernst equation: *E*_RHE_ = *E*_ref_ + (0.059 × pH), where *E*_ref_ = +0.104 V (measured for Hg/HgO electrode) and pH = 14 (measured for 1 M KOH). The samples used for testing were circular in shape with a diameter of 19 mm, yielding an active area of 2.83 cm^2^. The geometric area of the catalyst substrate was used to normalize the current density. No *iR* correction was performed to the obtained data. To prevent the accumulation of gas bubbles over the catalyst surface, the solution was continuously stirred during polarization and potentiostatic measurements. Anodic polarization tests were performed in the range of 0–1 V (vs Hg/HgO) with a scan rate of 2 mV/s and scanning from higher to lower potential. Prior to the polarization tests, the catalyst was conditioned by performing 30 cycles of cyclic voltammetry (CV) scans in the range of 0–0.8 V (vs Hg/HgO). The polarization measurements were repeated on multiple samples to ascertain the repeatability of results (Figure S1). Tafel slopes were obtained by linear fitting of the plot of log[*i*] versus the overpotential. Electrochemical impedance spectroscopy (EIS) was recorded in potentiostatic mode at a potential of 0.5 V (vs Hg/HgO) in the frequency range of 0.01 Hz to 2 MHz, with an amplitude of 25 mV. Electrochemical surface area (ECSA) was determined by calculating the double layer capacitance (*C*_DL_) from CV scans at different scan rates (100, 200, 300, 400, 500 mV/s) in the non-ohmic regime of 1.03 to 1.23 V. The difference in cathodic and anodic current densities (Δ*j*) at 1.15 V was plotted against the scan rates to calculate the *C*_DL_. Potentiostatic stability tests were performed at a potential of 1.58 V for 50 h, while the recycling tests were performed in the potential range of 0.05–0.7 V (vs Hg/HgO) for 2000 and 6000 cycles.

## 3 Results and Discussion

### 3.1 Surface Morphology and Structural Features

**Figure 1:**
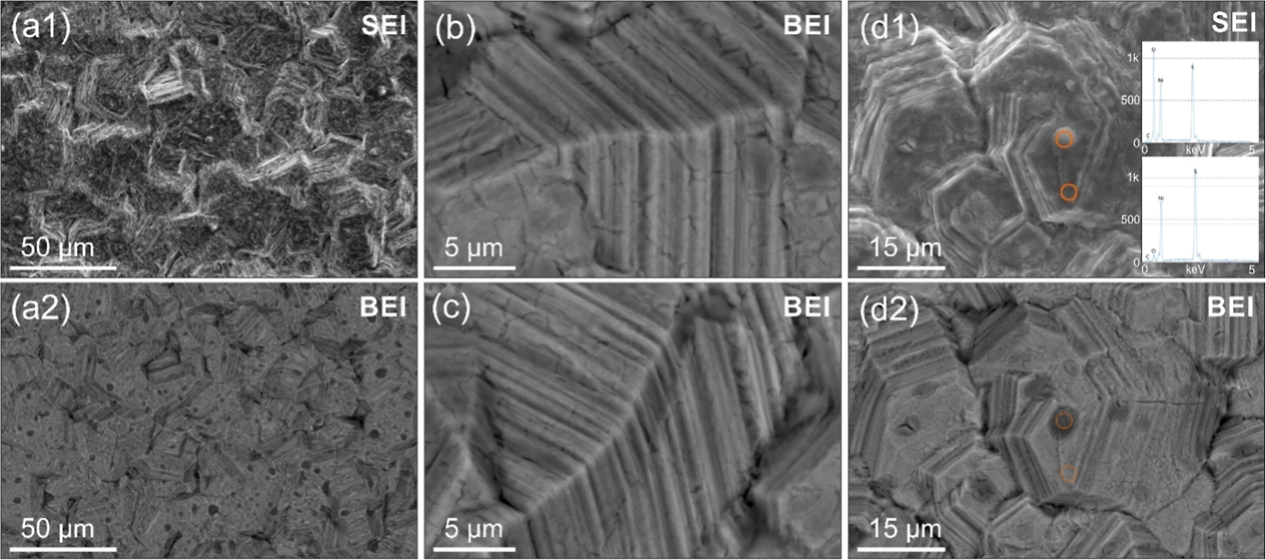
(a) SEM secondary electron (SE) and backscattered electron (BE) micrographs of NiS surface and morphology, with (b,c) details showing layered structure. (d) After exposure to air, darker spots on the surface show an oxygen-rich phase, as seen on corresponding EDS spectra (acquisition spots marked by circles, also presented in Figure S4).

The pristine Ni foil was used as-is, without further pretreatment besides thorough degreasing and washing. After the annealing and sulfurization process, the color of the nickel foil surface converted from silver to pale brass-yellow, indicating the formation of nickel sulfide on the surface. The altered samples were analyzed by SEM (Figure 1). The surface of the processed nickel foil featured a ∼1–2 μm thick layer (A tilted view of the image provided in Figure S2), which comprised densely packed 10–50 μm microcrystalline structures (Figure 1a), each comprised of 40–50 individual layers (Figure 1b,c). Upon prolonged exposure to air, the surface featured darker spots, 1–2 μm in size and visible on Z-sensitive backscattered electron (BE) micrographs. The chemical composition assessed by energy dispersive spectroscopy (EDS) shows an increased oxygen concentration, possibly due to the surface native oxide formation due to the exposure of nickel sulfide structures to ambient surroundings (Figure 1d). A detailed comparison of the chemical composition of grown nickel sulfide with the bare nickel foil is presented in Figure S3.

**Figure 2:**
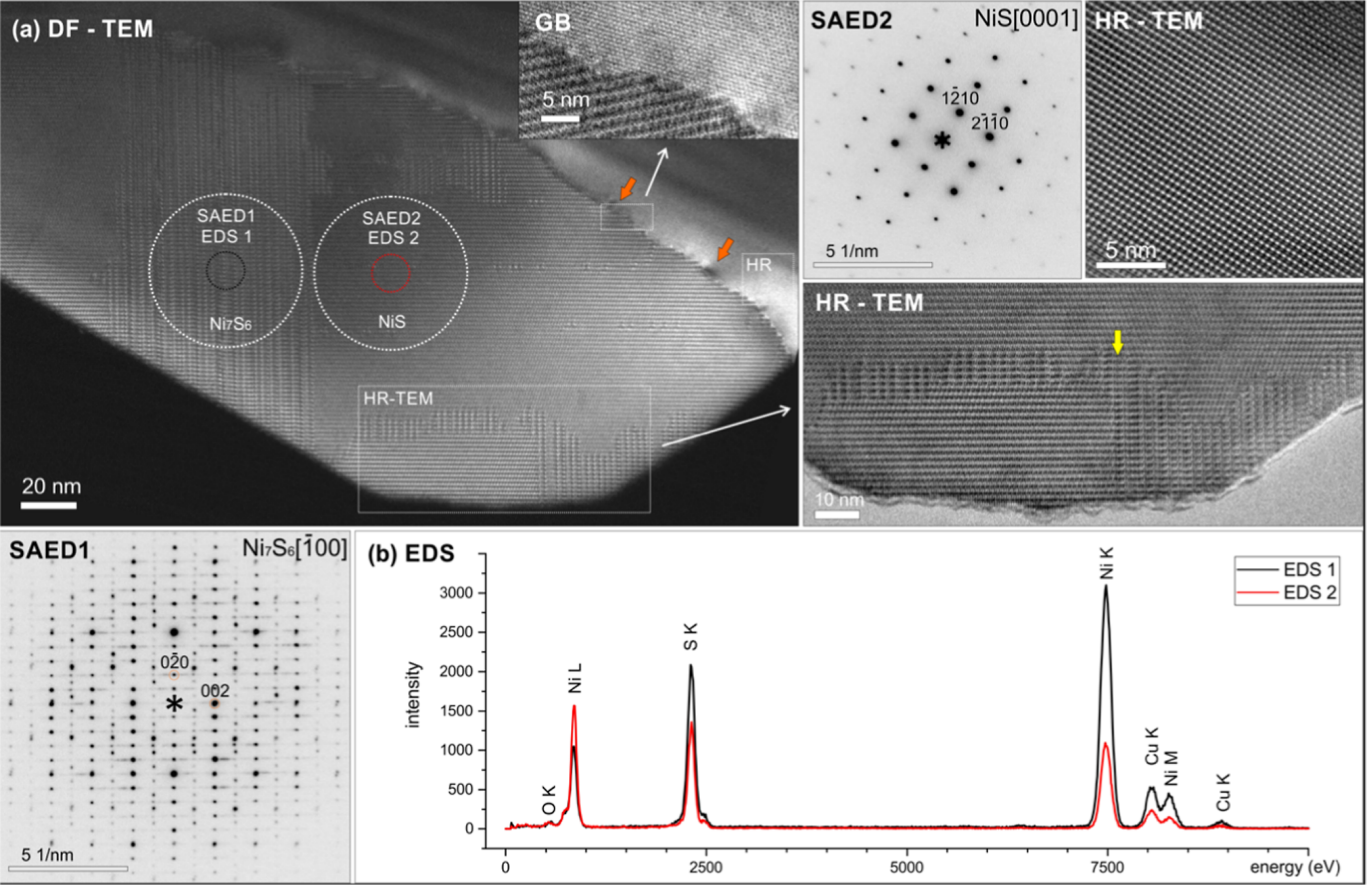
(a) Dark-field (DF) TEM micrograph of the 2 individual nickel sulfide crystals, with marked magnified regions and corresponding SAED. The orange arrows mark the large strain fields on the incoherent grain boundary interface, which is presented as a magnified region in the inset. The (b) EDS analysis positions are marked by circles and correspond to Ni-sulfides.

The crystal structure and phase composition of the microcrystallites were analyzed by TEM. Analyzed particulates are stable under the electron beam and consist of μm-sized firmly intergrown grains; the interface corresponds to an incoherent grain boundary. Inside individual grains, multiple epitaxially intergrown domains were identified. The structural analysis by selected-area electron diffraction (SAED) confirmed the presence of two nickel sulfide polymorphs—NiS and a non-stoichiometric Ni_7_S_6_. The analyzed representative single-crystal in Figure 2 shows regions of undisturbed, pristine NiS, with intergrown Ni_7_S_6_ lamellae in all low-index orientations. The point EDS analysis confirms a slight Ni surplus in the Ni_7_S_6_ regions, while a low amount of oxygen originates from superficial oxidation without Ni-oxide phases present in bulk; the extra Cu peak is an artifact from the Cu support grid. The SAED patterns recorded over the crystal correspond to an incommensurable structure originating from the nonperiodically intergrown few-atoms-thick layers of Ni_7_S_6_ in the NiS bulk.

**Figure 3:**
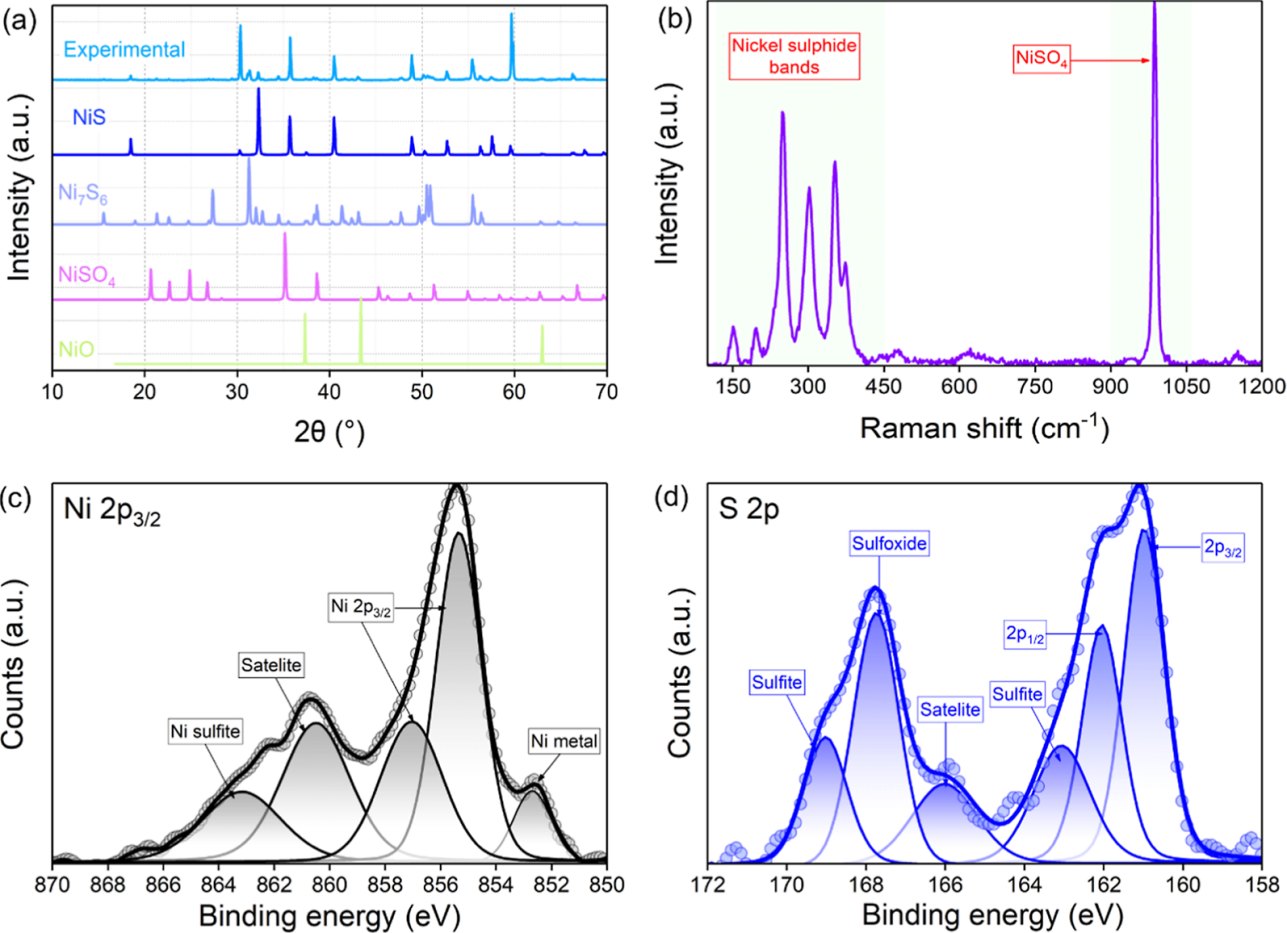
Crystal structure and chemical composition of the prepared NiS films. (a) XRD spectra, (b) Raman spectra, (c) high-resolution Ni 2p_3/2_ spectra and (d) high-resolution S 2p spectra.

The crystal structure and phase composition were assessed by X-ray diffraction (XRD); the experimental diffractograms and simulated patterns for millerite (NiS), Ni_7_S_6_ and NiSO_4_ are presented in Figure 3a. All the XRD peaks are labeled with corresponding *hkl* values after comparing them with the simulated XRD patterns (Table S1).^45^ The phase composition corresponds to a dual-phase nickel sulfide, NiS/Ni_7_S_6_, as observed by SAED. No Ni-oxide and NiSO_4_ matching phases were detected from the XRD spectra, confirming that the presence of oxygen is mainly only on the surface. Detailed information on the XRD peaks and crystal phases is given in Table S1.

The samples were analyzed using Raman spectroscopy to further confirm the structural properties of the nickel sulfide. The Raman spectra of the nanostructures were presented in Figure 3b, and the major bands observed correspond to the nickel sulfide bands and hydrous sulfate minerals. The nickel sulfide spectra consist of several bands centered at ∼150, 198, 248, 302, 353, and 373 cm^–1^. These peak positions are well-aligned with the reported ones for the NiS and Ni_7_S_6_.^46,47^ A sharp peak was observed at 985 cm^–1^, illustrating the presence of sulfate groups on the surface of the nickel sulfide that could correspond to the composition of the oxidized spots observed on SEM micrographs. All the structural and phase composition analyses suggest the presence of both the sulfide phases in the structure and minor oxide superficial features on the surface. Therefore, unravelling the surface constituents and chemical composition of the nanostructure is critical, and the sample was also examined by XPS. The XPS survey spectra of the nickel sulfide samples featured carbon, oxygen and nickel around 284.8, 532.2, and 850.2 eV, respectively, along with a peak of around 166.2 eV, corresponding to sulfur. The high-resolution Ni 2p and S 2p peaks were deconvoluted to identify the bonding configurations of the nanostructure. High-resolution Ni 2p spectra were deconvoluted into different peaks centered at 853.6, 856.1, 858.1, 861.3, and 864.2 eV (Figure 3c). The main peak observed at 856.1 eV and the satellite peak at 864.2 eV could be ascribed to surface oxides.^48,49^ The minor peaks at 853.6 eV suggest the presence of metallic nickel. The observation of Ni^3+^ 2p_3/2_ peak at 858.5 eV suggests bonding between the nickel and sulfur atoms.^45,50,51^ Moreover, the signal near 861.3 eV indicated the presence of an oxidation state of Ni^2+^.

The S 2p peak of the nanostructures suggested the presence of sulfide, sulfate, sulfite and sulfoxide components in the structure, as shown in Figure 3d. The doublet in the 163 eV region, deconvoluted into 161.8 and 163.2 eV, suggests the presence of higher-order nickel sulfides, indicating the presence of Ni_7_S_6_ in the compounds.^45^ The characteristic peaks at 169.8 and 163.2 eV confirm the existence of nickel sulfides. The sulfoxide peaks observed at 167.3 eV implied the presence of surface oxygen, as observed in Ni 2p spectra. In addition, the sulfate and sulfite peaks were visible at 168.5 and 169.8 eV, respectively, which aligns with the oxide formation noticed from the SEM and Raman analysis, which is more pronounced in the surface-sensitive XPS analysis. All these results were consistent with the dual-phase nickel sulfide (NiS and Ni_7_S_6_), as observed from the XRD and microscope analysis, with minor surface oxidation.

### 3.2 Electrocatalytic Measurements

**Figure 4:**
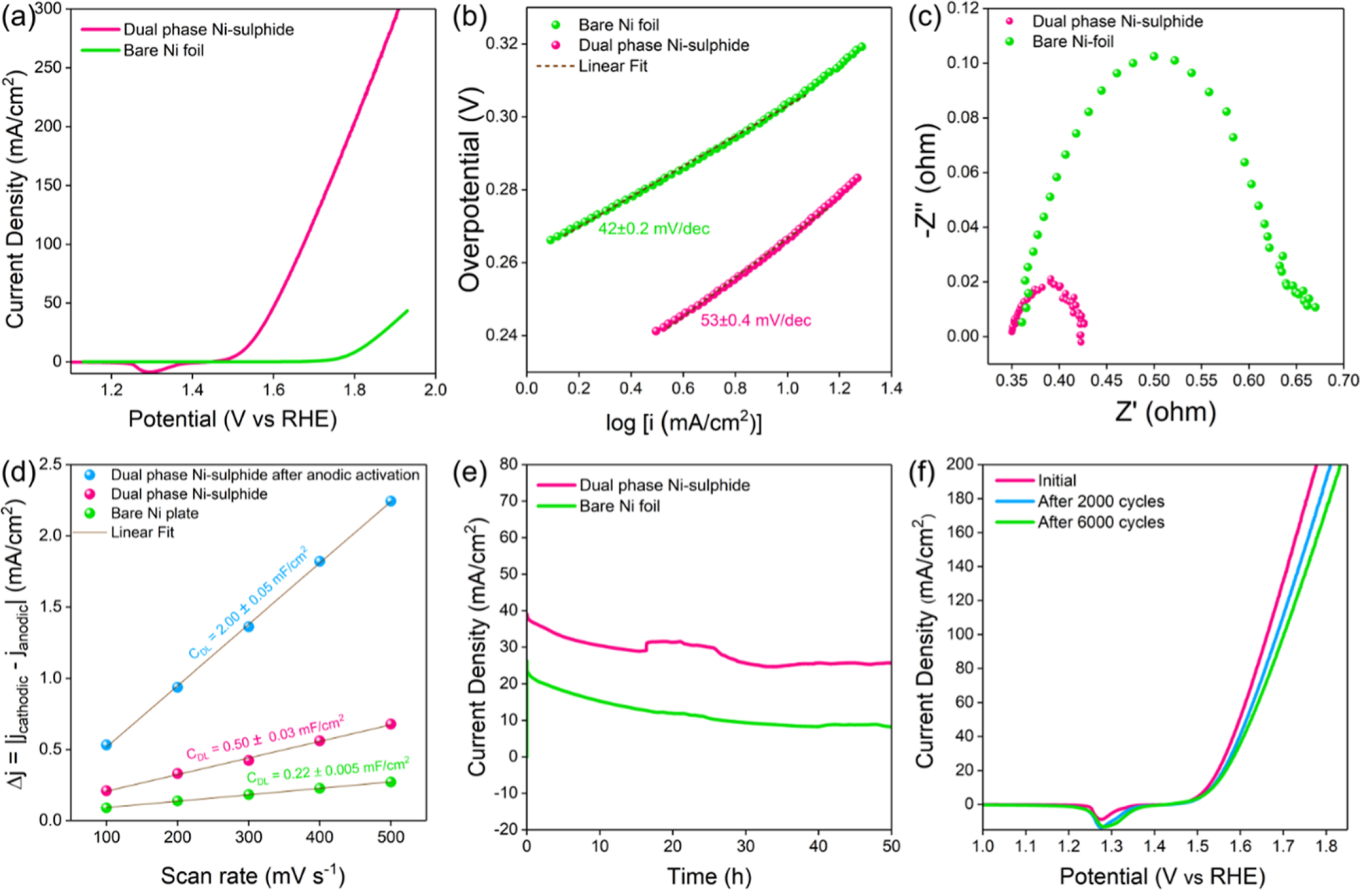
(a) Anodic polarization curve, (b) Tafel plots and (c) Nyquist plots obtained from EIS for bare Ni foil and dual phase Ni-sulfide in 1 M KOH; (d) plot representing the difference in cathodic and anodic current densities against the scan rate to determine *C*_DL_ and hence the ECSA; (e) 50 h potentiostatic test at 0.35 V overpotential for bare and dual phase Ni-sulfide; (f) anodic polarization curves for dual phase Ni-sulfide sample after 2000 and 6000 CV cycles.

The electrochemical performance of the dual-phase Ni-sulfide nanostructures was evaluated for OER by testing them in 1 M KOH electrolyte. Prior to activity measurements, the electrode surface was activated through multiple CV scans, and it was observed that the activity initially increased with subsequent scans and then stabilized. This behavior is commonly observed in non-oxide OER catalysts that are known to behave as a pre-catalyst and undergo surface reconstruction to form OER active species. The activity enhancement is manifested through linear anodic polarization data (Figure 4a), where the OER activity of the activated catalyst layer is much higher than that of the bare Ni substrate. For instance, bare Ni foil reaches the current density of 10 mA/cm^2^ at an overpotential (η_10_) of 0.58 V, and even when the applied overpotential is ∼0.70 V, the highest achievable current density is as low as ∼40 mA/cm^2^. Conversely, for the dual-phase Ni-sulfide layer, η_10_ reduces to a mere 0.29 V, while higher current densities of 100 and 300 mA/cm^2^ are obtained at overpotentials of 0.44 and 0.67 mV, respectively. This remarkable improvement in the OER activity is attributed to the activation of the dual-phase Ni-sulfide layer. The multiphase heterojunction and grain boundary defects, evident from the morphology analysis, also contribute to the adsorption and oxidation of reaction intermediates during OER. Tafel slopes for the bare and catalyst-coated foils are in a similar range of potentials (Figure 4b), suggesting similar OER kinetics. However, Nyquist plots (Figure 4c) obtained from EIS depict that the charge transfer resistance in dual phase Ni-sulfide (0.07 Ω) is almost 4 times smaller compared to that of the bare Ni foil (0.28 Ω). This further explains the better OER activity of dual-phase Ni-sulfide. Another crucial activity indicator is ECSA, which is indirectly determined by calculating the *C*_DL_ (Figures 4d and S5 and S6).

For bare Ni foil, *C*_DL_ is calculated to be 0.22 mF/cm^2^ and is more than doubled for dual-phase Ni-sulfide (0.50 mF/cm^2^). However, after anodic activation, the *C*_DL_ value increases almost 4 times (2.00 mF/cm^2^), suggesting that the surface transformation results in the creation of a large number of electroactive species that increase the ECSA and may assist in improving the OER rates, also reported in similar works.^52^ To assess their electrochemical stability, bare Ni foil and dual-phase Ni-sulfide catalysts were subjected to potentiostatic tests at an overpotential of 0.35 V for 50 h (Figure 4e). In the case of bare Ni foil, the current density fell by a whopping 35% in the first 10 h and another 46% in the next 40 h, substantiating its poor electrochemical stability. On the other hand, when the dual-phase Ni-sulfide was tested under similar potentiostatic conditions, the stability improved considerably, and the reduction in current density was halved (18%) in the first 10 h. In the next 40 h, the current density remains more or less stable, and a gradual drop of ∼16% is observed at the end of 50 h. The gradual decrease in stability is mainly due to the bubble pressure leading to catalyst layer detachment, as observed visibly during the reaction. The enormous number of bubbles generated during the measurement is presented in Movie S1. Furthermore, the recyclability of the dual-phase Ni sulfide was tested through continuous CV sweeps. At a current density of 100 mA/cm^2^, the overpotential increased marginally by 20 mV and 40 mV after 2000 and 6000 cycles of operation (Figure 4f), respectively, establishing the robustness of the catalyst for repeated industrial use. A comparison between the different nickel sulfide-based catalysts reported in the literature with the dual-phase Ni-sulfide fabricated in this research is presented in Table S2.^30,53−60^

### 3.3 Post-Mortem Analysis of the Electrodes

**Figure 5:**
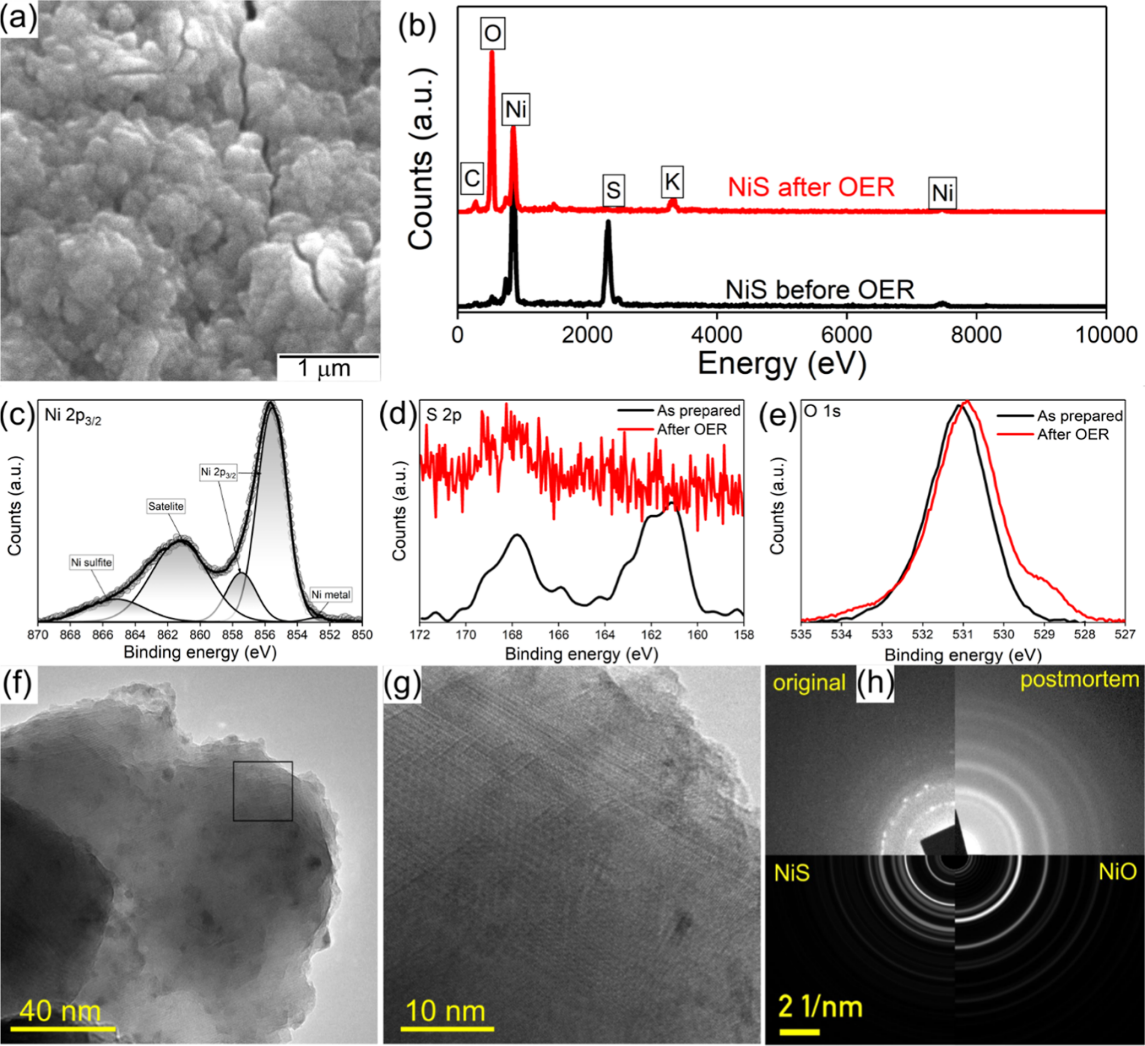
Surface and structural observation of dual-phase Ni-sulfide electrodes after OER experiments. (a) SEM micrograph of the surface, and (b) corresponding EDS spectra of the NiS samples before and after OER; (c) high-resolution Ni 2p spectra of Ni in NiS after OER; comparison of (d) high-resolution S 2p and (e) O 1s spectra of NiS electrodes. (f) Overview TEM micrograph of NiS electrode after OER, with enlarged inset (g) and pristine structure in the core still visible, covered by Ni-oxide. (h) Experimental SAED pattern of NiS electrodes before and after OER, compared to ab initio simulations for NiS and NiO.

The OER is mostly a surface reaction induced by the electrode/electrolyte interface that is responsible for the evolution of oxygen.^30^ Thus, the stability of heterogeneous polymorphs and crystallinity could significantly affect electrocatalysis. Therefore, the dual-phase nickel sulfide electrodes were further analyzed after the electrochemical testing to investigate the structural stability and possible chemical alterations. The SEM micrograph analysis shows the agglomeration of the surface, which could be due to the surface reconstruction during OER (Figure 5a). Therefore, the elemental analysis conducted by EDS on the surface and the spectra show a significant reduction of sulfur content, which agrees with the partial corrosion of sulfur observed prevalently in metal sulfides during OER.^61^ Metal sulfides are known to act as pre-catalysts, and their surface transforms into metal oxide-hydroxide structures during alkaline OER.^62^ This phenomenon was also observed for the dual-phase Ni-sulfide catalyst, as EDS showed a significant amount of oxygen in the sample after OER (Figure 5b). The observations were further confirmed by XPS analysis, revealing that the metallic Ni peak in the deconvolution of the Ni 2p peak of the catalyst after OER is reduced compared to pristine nanostructures (Figure 5c), and the sulfur peak is also reduced (Figure 5d). The XPS results also reveal that the oxygen peak is shifted, and the evolution of a new peak around 529.0 eV corresponds to the formation of metal oxide/hydroxide (Figure 5e), which is usually observed during OER. Deconvoluted O 1s spectra of pristine catalysts and after OER are presented in Figure S7. The peak area ratio of the metal oxide/hydroxide groups to the sulfur–oxygen groups changes from 3.9 to 8.2 after OER, indicating the transformation of the surface during the alkaline OER process. Besides, the deconvolution of peaks illustrates the shifting of NiO-related peaks and the changes in the area of NiO/Ni(OH)_*x*_, which could be due to the formation of NiOOH groups after the OER process. This indicates that the oxides/oxyhydroxide formation on the surface is dominated after OER. In addition to the surface and structural post-mortem analysis, the samples were characterized using TEM to identify the changes in the crystalline features of the materials.

The TEM micrographs illustrate numerous oxide-rich spherical regions, <5 nm in size, while most of the material is still dual-phase Ni-sulfide, and the oxidation is only superficial and localized.

TEM EDS mapping of the principal components after OER shows remaining Ni-sulfide in the core while the particles are completely covered by Ni-oxide, which originates from the oxide/hydroxide species (Figures S8 and S9). On comparing the SAED pattern of dual-phase NiS after OER, the crystal phases have not changed significantly. These results confirm that the transformation takes place only on the surface where sulfur gets corroded, and Ni is oxidized to higher states, forming NiOOH, consistent with the pre-catalyst theory proposed for non-oxide OER catalysts.^63^ As the core of the catalyst maintains the original dual nickel sulfide phase, it offers an easier charge conduction path due to its metallic nature, while the surface NiOOH facilitates the OER. This in-situ formed bilayer structure and the unique dual-phase structure provides an ideal catalyst composition to improve the OER kinetics, and the relatively lower charge transfer resistance enhances the oxygen evolution and boosts the water-splitting rate.

## 4 Conclusion

Using a low-temperature annealing method, a dual-phase Ni-sulfide nanostructure comprised of NiS and Ni_7_S_6_ phases was fabricated. The dual-phase Ni-sulfide nanostructure was directly grown on Ni-foil using H_2_S gas at 200 °C in the form of microcrystals composed of ∼50 individual layers. Excellent deposition uniformity was demonstrated in this method, and it also offers good adhesion between the nickel substrate to be directly used for the applications. As an electrocatalyst, dual-phase Ni-sulfide achieves 10 mA/cm^2^ at an overpotential (η_10_) of 0.29 V and an electrochemical stability for 50 h, exhibiting a comparable behavior to similar materials. The catalyst could withstand 6000 cycles of recycling, which suggests the robustness of the catalyst for practical industrial use. The post-mortem analysis of the catalyst material shows that the surface Ni-sulfide is transformed to NiOOH, which facilitates the OER, while the core maintains its metallic nature, assisting in easier charge conduction. The activation of the dual-phase heterojunctions of NiS/Ni_7_S_6_ material during the electrochemical catalysis provides numerous active sites for efficient OER activity, which could offer an important insight into the potential of dual-phase metal sulfides for water catalysis and, in turn, the affordable materials for the sustainable green hydrogen production.

## Supporting Information Available

Repeatability measurements; tilted SEM micrograph; chemical composition comparison; EDX spectra from different spots; additional CV testing; O 1s spectra; EDX mapping; performance comparison table (PDF)
The enormous number of bubbles generated during the measurement (Movie) (MOV)

## Supplementary Material

ef4c05182_si_001.pdf

ef4c05182_si_002.mov
