# Supplementary material for: Advancing Oxygen Evolution Catalysis with Dual-Phase Nickel Sulfide Nanostructures
Source: Energy Fuels. 2025 Jan 2;39(2):1375–83. doi: 10.1021/acs.energyfuels.4c05182 (PMC11748485; doi:10.1021/acs.energyfuels.4c05182)
Supplement: Supplementary file 1 — ef4c05182_si_001.pdf [file ef4c05182_si_001.pdf]

Supporting Information

Advancing Oxygen Evolution Catalysis with Dual Phase Nickel Sulfide Nanostructures

Neelakandan M Santhosh[1,2], Suraj Gupta[2,3], Vasyl Shvalya[1], Martin Košiček[1], Janez Zavašnik[1,4], Uroš Cvelbar[1,2*]*

[1]Department of Gaseous Electronics (F6), Jožef Stefan Institute, Jamova cesta 39,1000 Ljubljana, Slovenia

[2]Jožef Stefan International Postgraduate School, Jamova cesta 39, SI-1000 Ljubljana, Slovenia

[3]Advanced Materials Department, Jožef Stefan Institute, Jamova 39, 1000 Ljubljana, Slovenia

[4]Max-Planck-Institut für Nachhaltige Materialien, Max-Planck-Straße 1, 40237 Düsseldorf, Germany

*E-mail: neelakandan.m.santhosh@ijs.si, uros.cvelbar@ijs.si

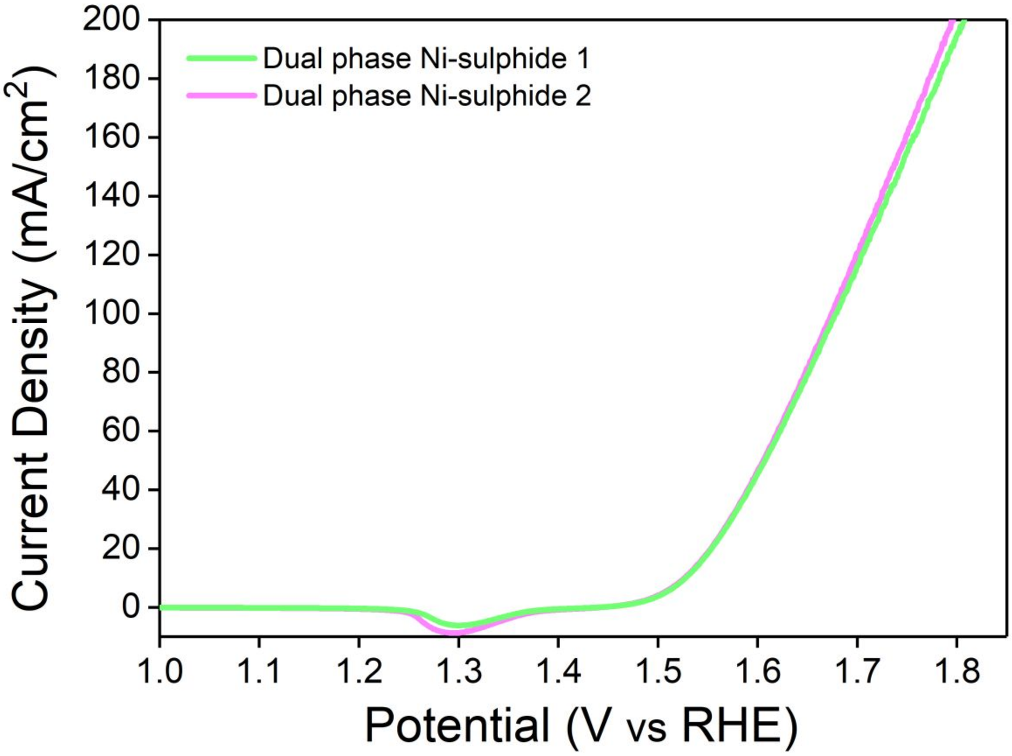

Figure S1. Anodic polarization curves for two different sets of Ni sulfide catalysts depict the repeatability of the measurements.

3

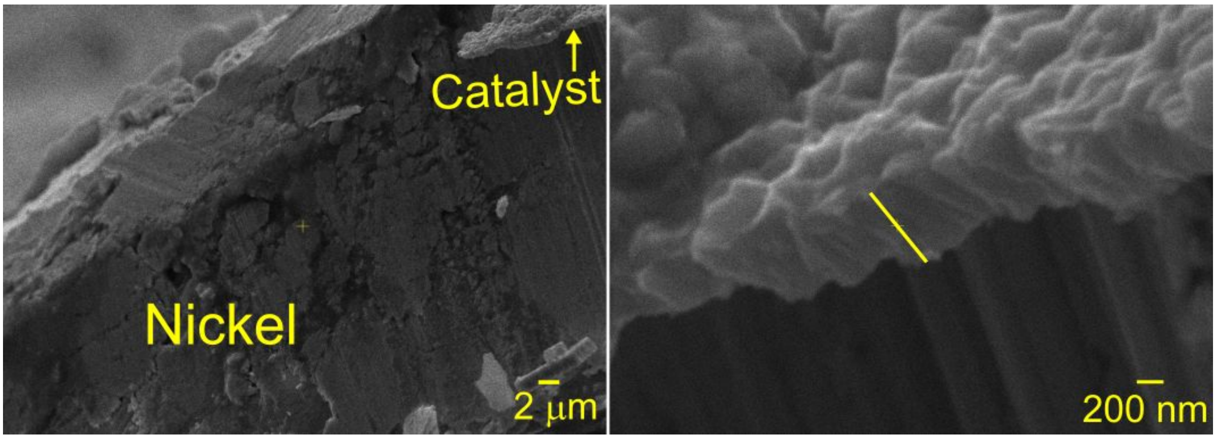

Figure S2. A tilted view of a dual-phase nickel sulfide catalyst to present the uniformly grown thin film and thickness of the thin film.

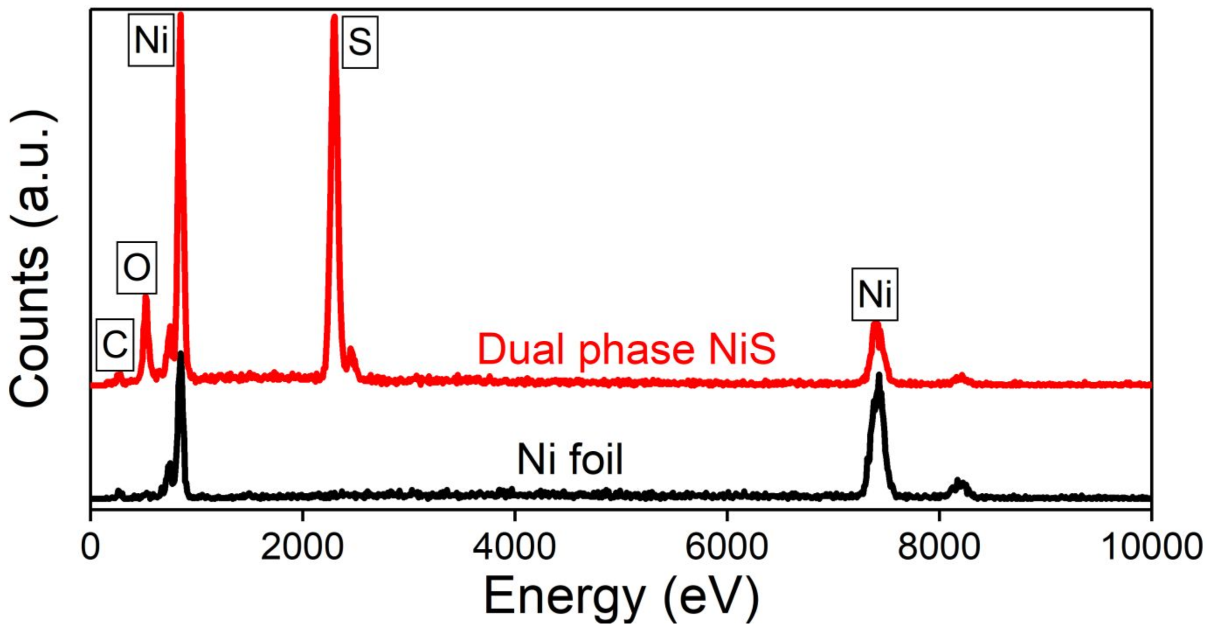

Figure S3. Chemical composition comparison of nickel sulfide with bare nickel foil.

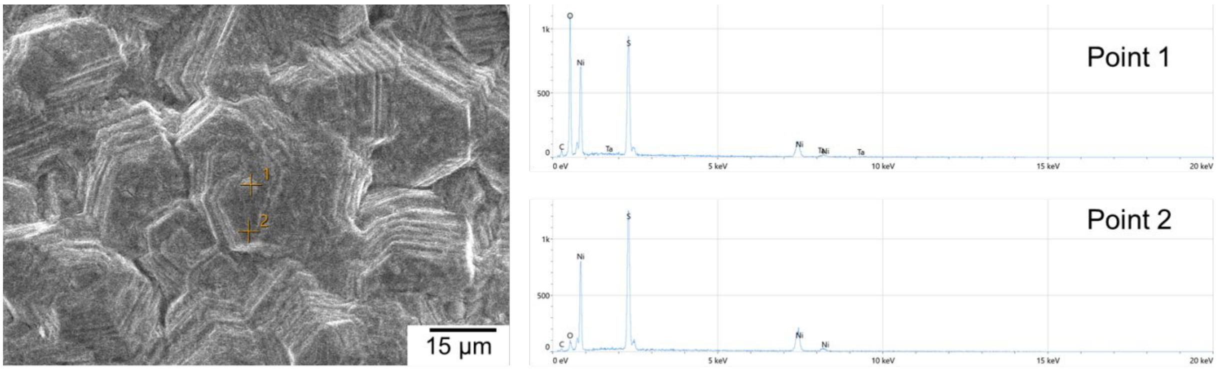

Figure S4. EDX spectra on different spots of nickel sulfide structures; darker spots on the surface show an oxygen-rich phase, as seen on corresponding EDS spectra (acquisition spots marked by circles)

XRD Analysis

Table S1. XRD patterns of the Dual Phase Nickel Sulfide nanostructures and corresponding *hkl* values

**Measured angle 2θ ~**	***hkl values of NiS***
18 °	(110)
30°	(101)
32°	(300)
35°	(220)
40°	(211)
49°	(131)
53°	(321)
56°	(330)
59°	(012)
67°	(122)

All the XRD peaks are labelled with corresponding *hkl* values after being compared with the simulated XRD patterns using structural data. Data for all CIF files used in the work:

- NiS (millerite): Grice, J. D., and R. B. Ferguson. "Crystal structure refinement of millerite (beta-NiS)." The Canadian Mineralogist 12.4 (1974): 248-252.
- Ni_3S_2 (heazlewoodite): Parise, John B. "Structure of heazlewoodite (Ni_3S_2)." Acta Crystallographica Section B: Structural Crystallography and Crystal Chemistry 36.5 (1980): 1179-1180.
- Ni_7S_6: Fleet, M. E. "The crystal structure of α-Ni_7S_6." Acta Crystallographica Section B: Structural Crystallography and Crystal Chemistry 28.4 (1972): 1237-1241.

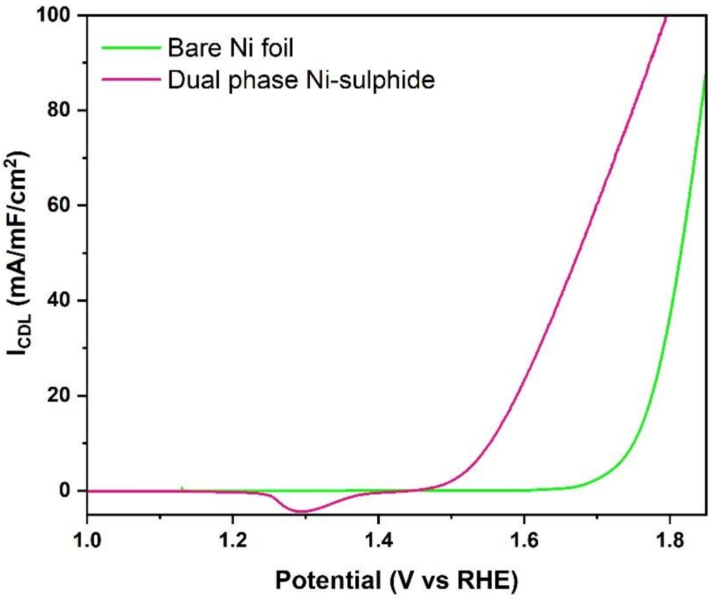

Figure S5. The normalized voltammogram data with the obtained C_{DL} values

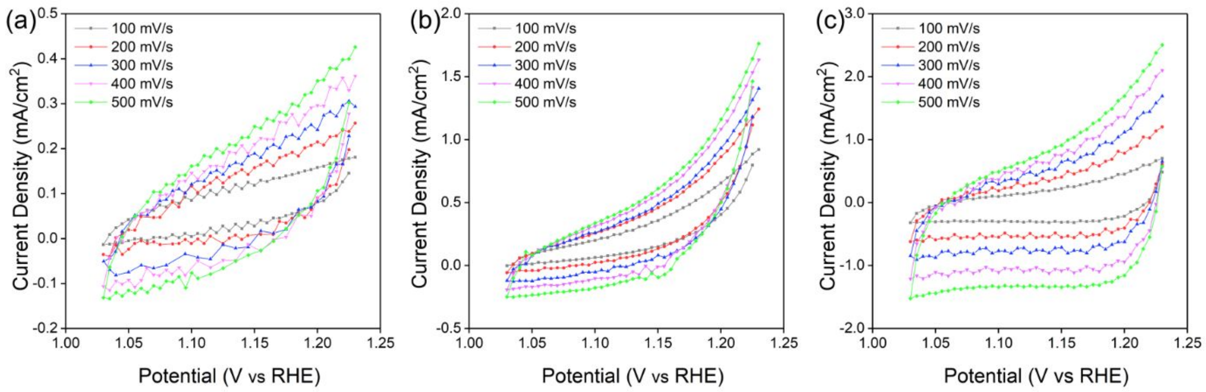

Figure S6. CV scans at increasing scan rates for **(a)** bare Ni foil, **(b)** pristine Ni sulfide and **(c)** Ni sulfide after anodic activation.

Post-mortem analysis of electrodes

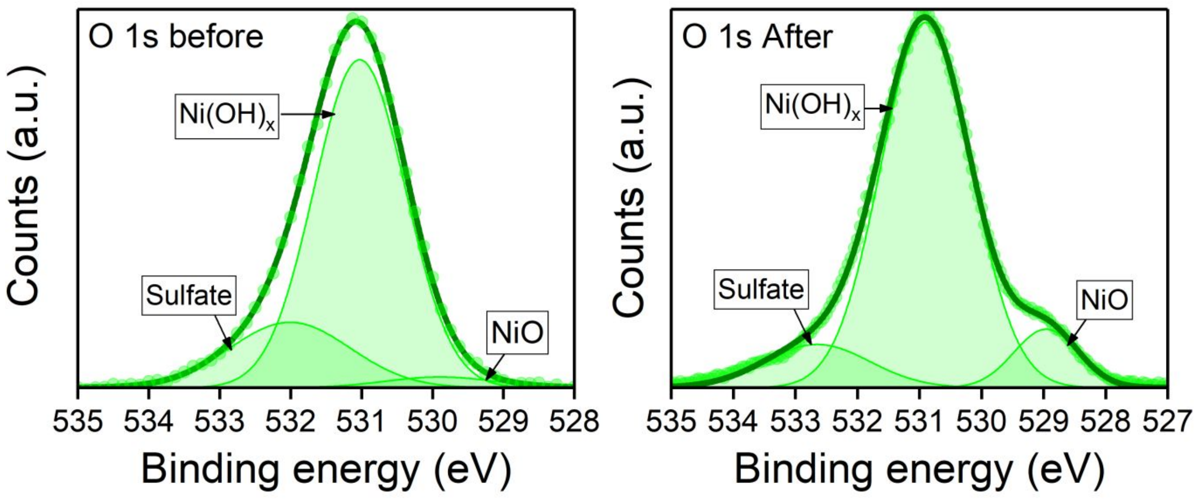

Figure S7. Deconvoluted O 1s spectra of the dual phase Ni-sulfide catalysts before and after OER experiments.

TEM EDS mapping of principal components for the dual-phase Ni sulfide sample after OES shows remaining Ni sulfide in the core while the particles are completely covered by Ni-oxide.

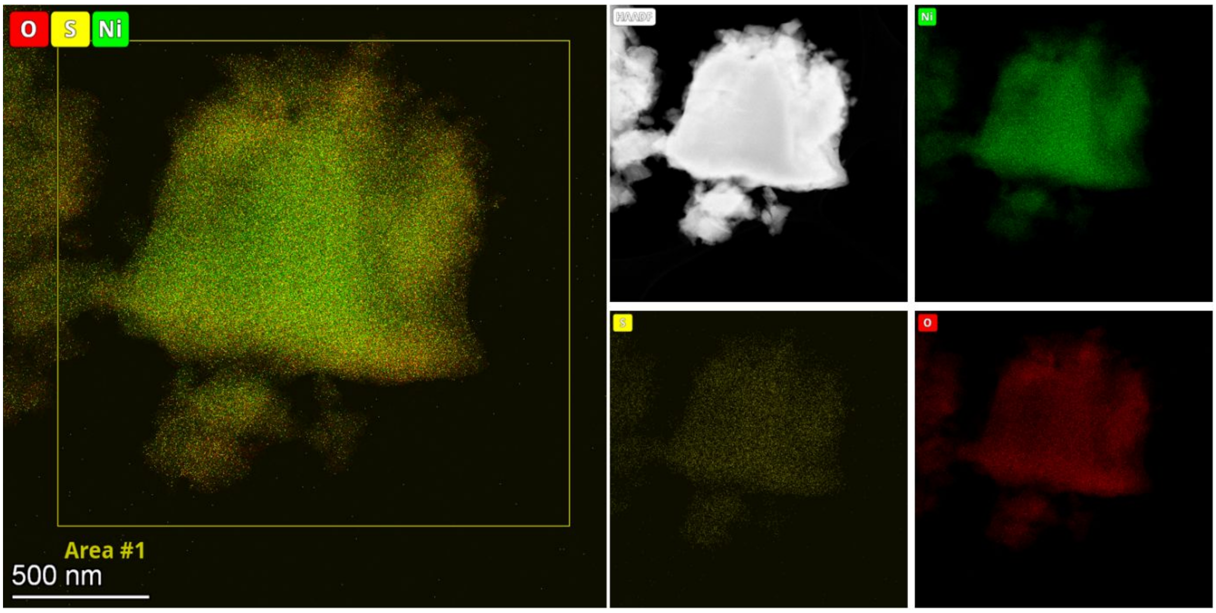

Figure S8. Ni-sulfide particle covered by Ni-oxide; composite picture and individual elemental maps, and corresponding HAADF-STEM micrograph.

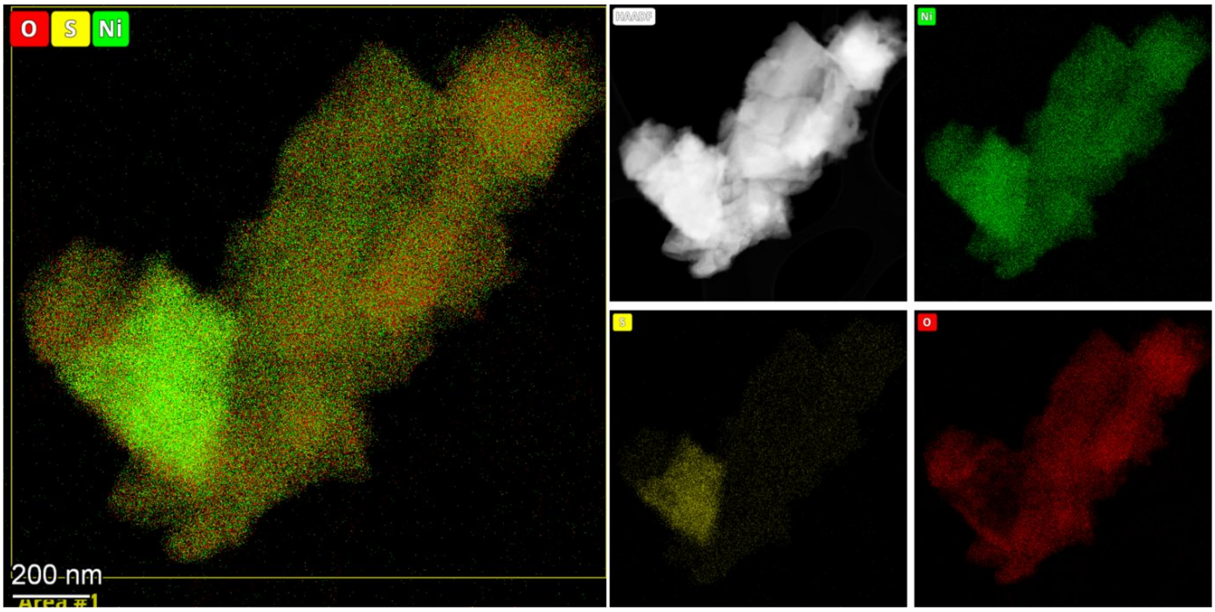

Figure S9. Unaltered and oxidized Ni-sulfide particles; composite picture and individual elemental maps; and corresponding HAADF-STEM micrograph.

Table S1. Comparison of OER catalytic performance of recently reported nickel sulfide polymorph catalysts.

Material	Electrolyte	Overpotential η (mV)	Current density (10 mA/cm^2)	Reference
Ni_9S_8/NixS nanoplates	1M KOH	329	10	1
Ni_xS_y (Multi Phase)	1M KOH	188	10	2
Ni_3S_2 foam/NF	1M KOH+ seawater	369	100	3
W–Ni_3S_2/Ni_7S_6	1M KOH	202	100	4
NiS/Ni foam	1M KOH	335	50	5
Ni_3S_2/Ni foam	1M KOH	312	10	6
N-NiS/NiS_2	1M KOH	270	10	7
NiS_2	1M KOH	362	20	8
Ni_3S_4	1M KOH	381	20	8
NiS	1M KOH	362	20	8
NiS	1M KOH	210	10	9
Dual-phase Ni-sulfide (NiS/Ni_7S_6)	1M KOH	290	10	**This work**

Reference

(1) Jiayi Chen; Xiaomin Xu; Rundong Mao; Cuifang Wang; Hsien-Yi Hsu; Zongyou Yin; A. Buntine, M.; Alexandra Suvorova; Martin Saunders; Zongping Shao; Guohua Jia. Strategic Cation Exchange Induced 2D Nickel Sulphide Nanoplates with Enhanced Oxygen Evolution Reaction Performance. *J Mater Chem A Mater* **2024**, *12* (40), 27364–27372. https://doi.org/10.1039/D4TA05191B.

(2) Khairy, M.; Liu, X.; Long, Z. Optimally Generated Active Sites on Nanostructured Nickel Sulfide Electrocatalysts for Designing Economical Electrolyzers. *ACS Appl Energy Mater* **2024**, *7* (14), 5822–5831. https://doi.org/10.1021/ACSAEM.4C00851/ASSET/IMAGES/LARGE/AE4C00851_0007.JPEG.

(3) Dai, Q.; He, X.; Yao, Y.; Dong, K.; Liu, X.; Guo, X.; Chen, J.; Fan, X.; Zheng, D.; Luo, Y.; Sun, S.; Li, L.; Chu, W.; Farouk, A.; Hamdy, M. S.; Sun, X.; Tang, B. Cauliflower-like Ni3S2 Foam for Ultrastable Oxygen Evolution Electrocatalysis in Alkaline Seawater. *Nano Res* **2024**, *17* (8), 6820–6825. https://doi.org/10.1007/S12274-024-6744-9/METRICS.

(4) Xue, Z.; Liu, Y.; Liu, Q.; Zhang, Y.; Yu, M.; Liang, Q.; Hu, J.; Li, G. Constructing Nickel Sulfide Heterojunctions by W-Doping-Induced Structural Transition for Enhanced Oxygen Evolution. *J Mater Chem A Mater* **2022**, *10* (7), 3341–3345. https://doi.org/10.1039/D2TA00149G.

(5) Zhang, X.; Zhang, S.; Li, J.; Wang, E. One-Step Synthesis of Well-Structured NiS–Ni2P2S6 Nanosheets on Nickel Foam for Efficient Overall Water Splitting. *J Mater Chem A Mater* **2017**, *5* (42), 22131–22136. https://doi.org/10.1039/C7TA05285E.

(6) Ren, G.; Hao, Q.; Mao, J.; Liang, L.; Liu, H.; Liu, C.; Zhang, J. Ultrafast Fabrication of Nickel Sulfide Film on Ni Foam for Efficient Overall Water Splitting. *Nanoscale* **2018**, *10* (36), 17347–17353. https://doi.org/10.1039/C8NR05494K.

(7) Liu, H.; Liu, Z.; Wang, F.; Feng, L. Efficient Catalysis of N Doped NiS/NiS2 Heterogeneous Structure. *Chemical Engineering Journal* **2020**, *397*, 125507. https://doi.org/10.1016/J.CEJ.2020.125507.

(8) Manjunatha, C.; Srinivasa, N.; Chaitra, S. K.; Sudeep, M.; Chandra Kumar, R.; Ashoka, S. Controlled Synthesis of Nickel Sulfide Polymorphs: Studies on the Effect of Morphology and Crystal Structure on OER Performance. *Mater Today Energy* **2020**, *16*, 100414. https://doi.org/10.1016/J.MTENER.2020.100414.

(9) Ehsan, M. A.; Khan, A.; Zafar, M. N.; Akber, U. A.; Hakeem, A. S.; Nazar, M. F. Aerosol-Assisted Chemical Vapor Deposition of Nickel Sulfide Nanowires for Electrochemical Water Oxidation. *Int J Hydrogen Energy* **2022**, *47* (100), 42001–42012. https://doi.org/10.1016/J.IJHYDENE.2021.10.231.
